# North-West London Hospital

**Published:** 1903-07-04

**Authors:** 


### NORTH-WEST LONDON HOSPITAL.

### SPECIAL APPEAL.

THE following appeal is being circulated by the authorities of the North-West London Hospital, Kentish Town Road:—

An urgent appeal for one million pennies to enable the treasurer, Mr. George Herring, to pay outstanding bills. One third of this amount has now been given. The fund is still open. It is hoped the remainder will soon be subscribed. Everbody can afford to give at least one penny, some can give silver, and others gold.

*Think of this*:—(1) Last year there were 601 in-patients and 43,674 attendances of out-patients. (2) It costs over £4,000 a year to carry on this work. (3) It is the most economically managed general hospital in London. (4) The report of King Edward's Hospital Fund says it is "well worthy of support." (5) It depends for support on free-will offerings of sympathising friends.

ALFRED CRASKE, Secretary.

That the hospital has obtained the hall-mark of approval by the Council of King Edward's Fund is the best argument in favour of its support, and one has only to take a walk through the wards to be convinced that the patients are thoroughly well cared for, and that the appliances and arrangements are as up-to-date as limited space and funds permit. Originated in 1872 by the persevering efforts of two ladies, Miss Learmonth (the Lady Superintendent) and her sister, the hospital has grown in scope and popularity, and is the only institution of the kind which serves the poor and densely-populated districts of Camden Town and Kentish Town, the line of demarcation between which runs through the building itself. The Helena wing, opened by H.R.H. Princess Christian in 1883, extends the accommodation to 53 beds, 18 being for children and 35 for adults. In one of the newer wards is a beautiful stained glass window in memory of members of Miss Learmonth's family who took a personal interest in the founding of the hospital. The theatre is well lighted and equipped with modern antiseptic appliances; the furniture both here and in the wards is of glass and white enamelled iron, and the operators' and anæsthetist's stools are of aluminium. There are special departments for diseases of the eye, skin and teeth, and for diseases of women. Accident and emergency cases are admitted at any time. There is a large out-patients' department. A recent addition is an outside staircase of iron, for use in case of fire.

The hospital is open for inspection daily, between the hours of 2 and 5 P.M., without any written order, and visitors are cordially welcomed.

